# Comparative Effects of Organic and Nano-Selenium on Egg Quality and Antioxidant Capacity in Layer Hens

**DOI:** 10.3390/foods14091454

**Published:** 2025-04-23

**Authors:** Yanhong Chen, Zhiqian Hao, Zengpeng Lv, Zhonghua Ning, Yanbin Guo, Jianmin Yuan

**Affiliations:** 1 College of Biological Science and Food Engineering, Southwest Forestry University, Kunming 650224, China; 2 State Key Laboratory of Animal Nutrition and Feeding, College of Animal Science and Technology, China Agricultural University, Beijing 100193, China; 3 National Engineering Laboratory for Animal Breeding, Department of Animal Genetics and Breeding, College of Animal Science and Technology, China Agricultural University, Beijing 100193, China; 4 College of Resources and Environmental Sciences, China Agricultural University, Beijing 100193, China

**Keywords:** nano-selenium, organic selenium, layer hens, yolk selenium deposition, antioxidant capacity

## Abstract

This study evaluates the effects of dietary selenium (Se) sources—sodium selenite (SS), nano-selenium (Nano-Se), selenocysteine (Se-C), and selenomethionine (Se-Met)—on production performance, egg quality, preservation characteristics, yolk Se content, and antioxidant capacity in Hy-Line Grey laying hens. A total of 450 healthy 18-week-old Hy-Line Grey laying hens were allocated to five groups (basal diet without Se, 0.30 mg/kg SS, Nano-Se, Se-C, or Se-Met) for an 8-week trial after a 4-week Se-depletion phase. The key results demonstrate that while no significant differences were observed in the feed intake, egg production rate, or egg weight among the groups (*p* > 0.05), organic Se (Se-C, Se-Met) and Nano-Se significantly improved the yolk color (*p* < 0.05) and yolk index (*p* < 0.05) and mitigated declines in the albumen height and Haugh unit during storage. Notably, Nano-Se exhibited superior efficacy in enhancing yolk color and antioxidant enzyme activity (*p* < 0.05). Furthermore, organic Se and Nano-Se increased yolk Se deposition (*p* < 0.05), increased yolk antioxidant enzyme activity (*p* < 0.05), and reduced lipid peroxidation (*p* < 0.05). These findings indicate that supplementing 0.3 mg/kg organic Se or Nano-Se enhances egg quality, extends shelf life, and improves antioxidant capacity, offering a sustainable strategy for selenium-enriched egg production.

## 1. Introduction

Selenium (Se) is an essential trace mineral for poultry health and development. Due to high egg production and increased metabolic activity, laying hens in peak production are particularly vulnerable to oxidative stress and are more susceptible to dietary Se deficiency [1,2]. This deficiency can lead to severe conditions, including exudative diathesis, white muscle disease, cerebellar degeneration, and pancreatic fibrosis [3,4,5,6].

However, the Se concentration in cereal grains is often insufficient to meet the minimum requirements for poultry [7,8]. As a result, inorganic Se, particularly sodium selenite (SS), is commonly used in poultry diets to prevent Se deficiency and support growth, reproduction, and overall health. Due to its potential toxicity at high levels, the European Union has set the authorized maximum value for supplementing inorganic Se at 0.3 mg/kg [9].

In past decades, alternatives to SS have been used, such as organic Se sources, including yeast selenium (SY), selenomethionine (Se-Met), and selenocysteine (Se-C), which are known for their high bioavailability [10,11]. Compared to inorganic Se, organic Se supplementation in laying hens could significantly increase egg Se content, enhance antioxidant capacity, promote intestinal development, and improve oviduct health [12,13,14]. Additionally, organic Se supplementation reduces drug residues, such as antibiotics, in the body and eggs [15].

Nano-selenium (Nano-Se) can enhance poultry production and maintain health due to its small particle size, high biological activity, strong antioxidant properties, and low toxicity [16]. Nano-Se regulates body metabolism, bolsters antioxidant defenses, and modulates intestinal microbiota flora, improving poultry health and growth performance [17,18]. It effectively mitigated the adverse effects of various pollutants on poultry muscle and liver tissues, resulting in higher-quality meat and other products for human consumption [19,20]. Our previous studies on broilers have also indicated that Nano-Se improved gut health and oxidative resilience [21,22], yet its impact on layer hens remains underexplored.

While prior research has emphasized Se’s role in egg enrichment, comparative analyses of organic and Nano-Se on egg preservation and quality parameters are limited. This study aimed to compare the effects of SS, Nano-Se, Se-C, and Se-Met on the production performance, yolk Se deposition, serum, and egg antioxidant capacity of laying hens, aiming to establish an optimal Se supplementation strategy for sustainable poultry production.

## 2. Materials and Methods

### 2.1. Animals and Experimental Design

A total of 450 healthy 18-week-old Hy-Line Grey layer hens were randomly assigned to five treatment groups, each consisting of five replicates with 18 hens. After being fed a basal diet without added Se for 4 weeks, the hens were divided and fed five diets with different Se sources: a control group (basal diet, no Se added), 0.30 mg/kg sodium selenite (SS), 0.30 mg/kg nano-selenium (Nano-Se), 0.30 mg/kg selenocysteine (Se-C), and 0.30 mg/kg selenomethionine (Se-Met). The SS (10,000 mg/kg) was brought from Guangzhou Yitong Biotechnology Co., Ltd., Guangzhou, China); the Nano-Se (30,000 mg/kg) was supplied by Beijing Wahmix Bio-Technology Co., Ltd., (Beijing, China); the Se-C (20,000 mg/kg) was brought from Angel Yeast (Chongzuo) Co., Ltd., (Chongzuo, China); the Se-Met (20,000 mg/kg) was brought from Hunan Yiheng Biological Technology Co., Ltd. (Changsha, China). The test period was 8 weeks.

The basal diet (Table 1) was formulated to meet the recommended nutrient requirements for Hy-Line Grey breeders, with the exception of Se. The analyzed Se concentrations in the diets are provided in Table 2. The hens were housed in two-story stepped cages, with environmental conditions (temperature, relative humidity, and lighting) following the Hy-Line Grey Management Guide. All experimental hens were caged in a 2-layer ladder-type cage, with each replicate cage evenly distributed in the barn. A combination of natural and artificial lighting was used, providing 16 h of light per day. During high summer temperatures, cooling was provided by wet curtain systems. The hens had unlimited access to food and water.

### 2.2. Sample Collection and Analytical Determination

#### 2.2.1. Laying Performance

The number of eggs and broken eggs and egg weights were recorded daily. The feed intake was recorded biweekly. The average egg weight, average daily egg production, incidence of egg breakage, average daily feed intake, and feed conversion rate were calculated every two weeks. The feed conversion ratio was calculated as the total feed intake (kg) divided by the total egg production (kg) from 18 hens per replicate.

#### 2.2.2. Egg Quality Determination, Preservation Performance, and Egg Se Assay

On weekends 2, 4, 6, and 8, 10 eggs per replicate were collected. Five eggs from every replicate were used to measure egg quality. The egg weight, shape index (ratio of short diameter to long diameter of eggs), yolk color (yolk color chart, Beijing Bullard Technology Development Co., Ltd., Beijing, China), albumen height and yolk height (protein height measuring instrument, Bulader-DA100, Beijing Bullard Technology Development Co., Ltd., Beijing, China), eggshell thickness (eggshell thickness tester, Bulader-M20, Beijing Bullard Technology Development Co., Ltd., Beijing, China), and yolk weight were determined. The yolk index and relative yolk weight were calculated.

Another five eggs from every replicate were stored under identical conditions. After storing for 5, 10, and 15 days, three eggs from every replicate were used to measure the albumen height, and the Haugh unit value was calculated.

Additionally, on these weekends, another set of three eggs per replicate was stored at room temperature (25 ± 3 °C) for 0, 5, 10, and 15 days before assessing the Haugh unit and albumen height. The egg yolks were mixed, digested with concentrated HNO_3_ (7697-37-2, Sinopharm Chemical Reagent Co., Ltd., Shanghai, China), and analyzed for Se content using hydride generation atomic fluorescence spectrometry [24].

#### 2.2.3. Antioxidant Capacity of Serum and Eggs

In the eighth week of the experiment, one hen from every replicate was used for blood collection (5 mL) from the wing vein. The blood was centrifuged for 10 min, and the serum was collected and stored at −20 °C for subsequent analysis.

Biochemical and antioxidant indices in the serum and yolk were determined using assay kits purchased from Nanjing Jianzheng Reagent Co., Ltd. (Nanjing, China).

The concentrations of total protein (TP), albumin (ALB), glutathione peroxidase (GSH-Px), catalase (CAT), and malondialdehyde (MDA) were assessed using kits from Nanjing Jiancheng Bioengineering Institute (catalog numbers: A045-2-2, A028-1-1, A005-1-2, A007-1-1, and A003-1-1).

#### 2.2.4. Statistical Analysis

Statistical analysis was performed using SPSS 21.0 software. Prior to conducting the one-way analysis of variance (ANOVA), the data normality was tested using Levene’s test for homogeneity of variances. ANOVA was conducted, and Duncan’s multiple comparison method was applied to identify significant differences among the groups. The data are presented as the mean ± standard error of the mean (SEM). Differences were considered statistically significant at *p* < 0.05.

## 3. Results

### 3.1. Production Performance

Dietary supplementation with different Se sources had no significant difference on the average feed intake, feed conversion ratio, egg production rate, average egg weight, or broken egg rate at weeks 2, 4, 6, 8, and 0–8 (*p* > 0.05; Table 3).

### 3.2. Egg Quality

Dietary supplementation with Se affected the egg shape, and 0.30 mg/kg SS, 0.30 mg/kg Se-C, and 0.30 mg/kg Se-Met significantly decreased the egg shape index (*p* < 0.05; Table 4) at weeks 0–8. There were no significant differences among the 0.30 mg/kg SS, 0.30 mg/kg Se-C, and 0.30 mg/kg Se-Met groups (*p* > 0.05).

Dietary supplementation with Se significantly increased the yolk color at weeks 0–8 (*p* < 0.05). Moreover, supplementation with 0.30 mg/kg Nano-Se or 0.30 mg/kg Se-C significantly increased the yolk color compared to 0.30 mg/kg SS or 0.30 mg/kg Se-Met supplementation (*p* < 0.05).

Dietary supplementation with Se significantly increased the yolk height at weeks 0–8 (*p* < 0.05), and 0.30 mg/kg Nano-Se increased the yolk height (*p* < 0.05). However, there were no significant differences among the 0.30 mg/kg Se-C, 0.30 mg/kg Se-Met, and Con groups (*p* > 0.05).

Dietary supplementation with Se could significantly affect the yolk index at weeks 0–8, and the yolk index was significantly greater in the 0.30 mg/kg Nano-Se group compared with the 0.30 mg/kg SS and 0.30 mg/kg Se-Met groups (*p* < 0.05). However, there were no significant differences among the 0.30 mg/kg SS, 0.30 mg/kg Se-Met, and 0.30 mg/kg Se-C groups (*p* > 0.05).

Dietary supplementation with 0.30 mg/kg Se-C and 0.30 mg/kg Se-Met increased the relative weight of egg yolk (*p* < 0.05) at weeks 0–8 compared to the control. However, there were no significant differences among the 0.30 mg/kg SS, 0.30 mg/kg Nano-Se, 0.30 mg/kg Se-C, and 0.30 mg/kg Se-Met groups (*p* > 0.05).

### 3.3. Preservation Performance

In the second week of the experiment, after the eggs were stored for 10 days, compared to the Con group, dietary supplementation with Se increased the albumen height and Haugh unit (*p* < 0.05; Table 5). There were no significant differences among the Se groups (*p* > 0.05).

In the fourth week of the experiment, after the eggs were stored for 15 days, compared to the Con group, dietary supplementation with Se tended to increase the albumen height and Haugh unit of the eggs (0.05 < *p* < 0.01).

In the sixth week of the experiment, after the eggs were stored for 0 days, compared to the Con group, dietary supplementation with Se tended to increase the albumen height (0.05 < *p* < 0.01). Dietary supplementation with Se increased the Haugh unit of the eggs (*p* < 0.05). There were no significant differences among the Se groups (*p* > 0.05).

In the eighth week of the experiment, after the eggs were stored for 0 day, compared to the Con group, dietary supplementation with 0.30 mg/kg Se-C and 0.30 mg/kg Se-Met increased the albumen height and Haugh unit of the eggs (*p* < 0.05). Moreover, the albumen height and Haugh unit of the eggs were significantly higher with the 0.30 mg/kg Se-Met supplementation than with the 0.30 mg/kg Se-C supplementation (*p* < 0.05). After the eggs were stored for 5 days, dietary supplementation with Se tended to increase the albumen height (0.05 < *p* < 0.01).

### 3.4. Egg Se Content

Dietary supplementation with Se increased the egg Se content from 2 wks, compared to the Con group, (*p* < 0.05; Table 6). However, there were no significant differences among the Se groups (*p* > 0.05).

### 3.5. Antioxidant Capacity of the Serum and Yolk

Dietary supplementation with Se increased the GSH-Px content in the serum and yolk compared to the Con group (*p* < 0.05; Table 7). There were no significant differences among the Se groups (*p* > 0.05). Dietary supplementation with 0.30 mg/kg Nano-Se, 0.30 mg/kg Se-C, and 0.30 mg/kg Se-Met decreased the MDA content in the serum and yolk (*p* < 0.05; Table 7); however, there were no significant differences among the 0.30 mg/kg Nano-Se, 0.30 mg/kg Se-C, and 0.30 mg/kg Se-Met groups (*p* > 0.05).

## 4. Discussion

Dietary Se can enhance poultry production performance. However, the comparative effects of various Se sources, such as SS, Nano-Se, Se-C, and Se-Met, on the performance of laying hens have rarely been investigated. Our study demonstrated that different Se sources resulted in no significant differences in the average feed intake, feed conversion ratio, egg production rate, average egg weight, or broken egg rate. These findings are consistent with previous studies [25,26] and may be attributed to the sufficient basal antioxidant capacity in young hens. In addition, Se in the basal diet might meet the growth and development needs of layer hens.

Consumers in Northeast China have a preference for eggs with darker yolks [27]. Our results firstly indicate that 0.30 mg/kg Nano-Se increased the yolk color. Similarly, dietary Se supplementation improved the egg yolk color [28], and this effect was also observed under heat stress conditions [29]. Due to the absence of yolk color measurements using a precise colorimeter, the relevant literature on antioxidants was referenced to support these findings. The yolk color is mainly affected by the oxycarotenoids in the diet. When oxycarotenoids are oxidized, the pigment intensity becomes weaker [30]. The egg yolk color was significantly positively correlated with antioxidant enzyme levels and Se content in the egg yolk, while it was significantly negatively correlated with MDA levels [30]. The Se content in eggs is highly correlated with the Se content in feed [31], and the deposition in eggs can protect the egg shell and improve the antioxidant capacity of egg yolk [32]. The subsequent antioxidant capacity tests of the serum and yolk also indicate that Nano-Se might act as an internal antioxidant component of the yolk to regulate the oxidation process of the yolk, promote the accumulation of xanthophylls, and improve the yolk color.

Egg freshness is also a parameter highly valued by consumers. The egg white height, yolk height, Haugh unit, and yolk index are technical indicators that can objectively reflect the freshness of eggs [33]. This study demonstrated that the reduction in egg albumen height and Haugh unit occurred most rapidly during the initial 5 days of storage, after which the rate of decline gradually decelerated. Organic Se or Nano-Se significantly mitigated the decrease in egg protein height and Haugh unit. Furthermore, dietary supplementation with organic Se or Nano-Se for over 6 weeks resulted in an elevated Haugh unit value and extended the shelf life of the eggs. Chen et al. [32] and Mohammadsadeghi et al. [30] also found that the egg white height and Haugh unit were significantly higher in the organic Se or Nano-Se groups. During storage, egg proteins undergo oxidative degradation, leading to a decreased total sulfhydryl content, increased carbonyl content, and a significant rise in malondialdehyde (MDA) levels over time [34,35]. Selenium can reduce MDA and carbonyl levels, inhibit the oxidative attack of reactive oxygen species (ROS) on egg yolk proteins, and enhance the antioxidant capacity [36]. Additionally, selenium is reduced to selenocysteine in animals and binds to proteins. High doses of Se are beneficial for improving protein quality, and as Se accumulates in eggs, the corresponding protein quality improves [37]. Selenium can act as an antioxidant to protect unsaturated fatty acids, which may help maintain the structural integrity of eggs, particularly the membrane structure [38]. In the producing and marketing of egg products, selenium is beneficial for maintaining egg quality and offers commercial advantages during egg transportation and storage. Therefore, maternal dietary supplementation with 0.3 mg/kg organic Se or Nano-Se can maintain egg quality during storage and enhance the commercial value of eggs.

Dietary selenium supplementation can enhance the selenium content in eggs [26]. The Se concentration in eggs depends on the dietary Se level and its source form [9]. Experimental results have indicated that organic forms of Se exhibit greater bioavailability compared to inorganic forms, which is reflected in higher selenium deposition in eggs [37,39]. Our results indicate that Nano-Se, Se-C, and Se-Met increased the egg Se content. Organic Se is more efficiently deposited into eggs compared to inorganic Se [40]. Increasing the amount of Se in a laying hen’s diet increases the Se content in eggs [41]. It is plausible that organic Se and Nano-Se participate in the protein synthesis process in laying hens. During egg formation, selenium is integrated into histones, elevating the Se content in eggs. After feeding 0.3 mg/kg yeast Se to laying hens, the Se content of eggs can reach the standard of 0.3 mg/kg Se-rich eggs [13,14]. However, no significant differences in egg yolk selenium content were observed across different sampling points. This may be related to the physiological stage of the laying hens and the fact that albumen selenium content was not measured.

Egg quality also is closely related to the antioxidant capacity of the egg and layer hens. Selenium is a constituent of selenocysteine, the active center of glutathione peroxidase [42]. As an important peroxidolytic enzyme, glutathione peroxidase (GSH-Px) catalyzes the conversion of reduced glutathione (GSH) to oxidized glutathione (GSSG) and reduces toxic peroxides to non-toxic hydroxyl compounds [43]. Organic Se is more readily absorbed and incorporated into tissues compared to inorganic forms, leading to higher Se concentrations and consequently greater GSH-Px activity [10,37]. In this experiment, maternal dietary supplementation with Nano-Se, Se-C, and Se-Met increased the GSH-Px content in the serum and yolk, and also decreased the MDA content in the serum and yolk, which is consistent with a previous study [29]. Lipid peroxides are oxidized to form the end product MDA, which shows the degree of damage of body lipids attacked by reactive oxygen free radicals [44]. Organic Se and Nano-Se increased the total antioxidant capacity (T-AOC) content and reduced the MDA content in egg yolk [32,37,45]. It may be that the addition of organic Se or Nano-Se increases the Se content in egg yolk and laying hens, enhancing the activity of selenium-related antioxidant enzyme, while also boosting the antioxidant capacity of the hens.

## 5. Conclusions

Supplementation with 0.30 mg/kg Nano-Se significantly improves yolk Se deposition, antioxidant capacity, and egg preservation without compromising production performance, offering a sustainable strategy for selenium-enriched egg production.

## Figures and Tables

**Table 1:** Ingredient composition of the basal diets.

Ingredients	%	Nutrient Composition	%
Corn	57.00	ME (kcal/kg)	2.70
Soybean meal	29.50	CP	17.30
Soybean oil	1.50	Lysine	0.87
Shell powder	7.00	Methionine	0.36
Calcium hydrogen phosphate	0.85	Cysteine	0.65
Table salt	0.35	Threonine	0.66
Trace mineral premix ^1^ (no sodium selenite)	0.20	Tryptophan	0.21
Vitamin premix ^2^	0.03	Arginine	1.07
L-Choline chloride (50%)	0.10	Isoleucine	0.70
Phytase (10,000 U/g)	0.02	Calcium	4.04
DL-Methionine (98%)	0.12	Non-phytate phosphorus	0.36
Fine stone powder	3.33		
Total	100.00		

^1^ The trace mineral premix provided the following per kg of diets: Fe: 67.20 mg; Cu: 6.40 mg; Zn: 55.20 mg; Mn: 68.66 mg; I: 0.72 mg; P: 36.55 g. ^2^ The vitamin premix provided the following per kg of diets: vitamin A: 7709 IU; vitamin E: 60 IU; vitamin D_3_: 2200 IU; vitamin K_3_: 9 mg; vitamin B_1_: 2.75 mg; vitamin B_2_: 7.7 mg; pantothenic acid: 33 mg; vitamin B_12_: 0.044 mg; biotin: 0.22 mg; niacin: 33 mg; choline: 287 mg.

**Table 2:** Supplemented and analyzed levels of selenium in dietary treatments (mg/kg).

Treatment	Supplemented Values		Analyzed Values ^1^
	Sources	Levels	Diets
A	No additional Se	0.00	0.13
B	SS	0.30	0.51
C	Nano-Se	0.30	0.54
D	Sec	0.30	0.47
E	SeMet	0.30	0.52

^1^ Selenium concentrations of dietary treatments were analyzed by hydride generation atomic absorption spectrophotometry [23].

**Table 3:** Effects of different Se sources on production performance of laying hens.

Items	Time	Con	0.30 mg/kg SS	0.30 mg/kg Nano-Se	0.30 mg/kg Se-C	0.30 mg/kg Se-Met	SEM	*p*-Value
Average feed intake (g)	2 wks	103.11	105.64	102.31	105.02	102.91	0.471	0.096
	4 wks	110.31	110.14	110.57	108.77	109.54	0.311	0.389
	6 wks	116.65	117.75	118.28	117.34	116.87	0.501	0.869
	8 wks	115.51	116.54	117.04	116.58	116.72	0.300	0.600
	0–8 wks	111.40	112.52	112.05	111.93	111.51	0.160	0.170
Feed conversion ratio	2 wks	2.25	2.24	2.18	2.20	2.27	0.021	0.710
	4 wks	2.22	2.17	2.17	2.16	2.14	0.025	0.904
	6 wks	2.27	2.29	2.31	2.28	2.27	0.028	0.991
	8 wks	2.10	2.11	2.13	2.14	2.10	0.018	0.919
	0–8 wks	2.21	2.20	2.20	2.20	2.19	0.014	0.995
Egg production rate (%)	2 wks	86.11	88.02	86.83	89.21	86.59	0.831	0.799
	4 wks	90.71	90.40	92.30	90.16	90.71	0.868	0.955
	6 wks	89.13	90.19	90.24	90.47	91.11	0.969	0.984
	8 wks	92.86	93.09	93.81	93.60	94.17	0.719	0.983
	0–8 wks	89.70	90.42	90.79	90.86	90.65	0.625	0.983
Average egg weight (g)	2 wks	53.25	53.75	54.03	53.67	52.62	0.256	0.484
	4 wks	55.10	56.25	55.50	55.94	56.76	0.633	0.947
	6 wks	57.70	57.14	56.89	57.20	56.94	0.144	0.437
	8 wks	59.34	59.52	58.52	58.14	59.25	0.205	0.144
	0–8 wks	56.35	56.67	56.24	56.24	56.39	0.188	0.961
Broken egg rate (%)	2 wks	3.27	2.42	2.25	1.96	2.57	0.296	0.736
	4 wks	2.63	2.22	2.25	1.89	3.24	0.291	0.674
	6 wks	2.55	2.54	2.10	2.97	2.83	0.290	0.917
	8 wks	2.57	2.25	2.95	3.34	2.59	0.241	0.696
	0–8 wks	2.75	2.36	2.39	2.54	2.80	0.163	0.887

Note: Means in a column with different letters are significantly different (*p* < 0.05).

**Table 4:** Effects of different Se sources on egg quality of laying hens.

Items	Time	Con	0.30 mg/kg SS	0.30 mg/kg Nano-Se	0.30 mg/kg Se-C	0.30 mg/kg Se-Met	SEM	*p*-Value
Egg shape index	2 wks	1.29	1.32	1.35	1.31	1.29	0.007	0.062
	4 wks	1.40 ^a^	1.30 ^b^	1.29 ^b^	1.30 ^b^	1.29 ^b^	0.011	<0.001
	6 wks	1.29	1.30	1.29	1.28	1.30	0.004	0.425
	8 wks	1.29	1.30	1.31	1.31	1.30	0.004	0.663
	0–8 wks	1.32 ^a^	1.30 ^b^	1.31 ^ab^	1.30 ^b^	1.30 ^b^	0.003	0.048
Yolk color	2 wks	7.60 ^b^	8.32 ^a^	7.90 ^ab^	8.32 ^a^	7.88 ^ab^	0.086	0.017
	4 wks	7.68 ^c^	8.20 ^b^	8.64 ^a^	8.52 ^ab^	8.40 ^ab^	0.088	0.001
	6 wks	7.52 ^b^	7.48 ^b^	8.20 ^a^	8.08 ^a^	7.60 ^b^	0.083	0.002
	8 wks	8.00	8.00	8.12	7.88	7.88	0.063	0.757
	0–8 wks	7.70 ^c^	8.00 ^b^	8.22 ^a^	8.20 ^a^	7.94 ^b^	0.047	<0.001
Yolk height (mm)	2 wks	14.18	14.31	14.70	14.72	14.22	0.102	0.252
	4 wks	14.92	14.83	15.33	14.94	15.13	0.075	0.214
	6 wks	14.21 ^b^	15.08 ^a^	15.06 ^a^	14.46 ^ab^	14.72 ^ab^	0.106	0.022
	8 wks	15.37	15.08	15.38	15.49	15.31	0.080	0.617
	0–8 wks	14.67 ^b^	14.83 ^b^	15.12 ^a^	14.90 ^ab^	14.85 ^b^	0.044	0.015
Yolk index	2 wks	0.41 ^c^	0.43 ^abc^	0.45 ^a^	0.44 ^ab^	0.42 ^bc^	0.004	0.027
	4 wks	0.45	0.44	0.44	0.45	0.46	0.004	0.877
	6 wks	0.38 ^c^	0.41 ^ab^	0.42 ^a^	0.39 ^bc^	0.40 ^abc^	0.004	0.016
	8 wks	0.40	0.40	0.42	0.42	0.42	0.004	0.086
	0–8 wks	0.41 ^c^	0.42 ^b^	0.43 ^a^	0.43 ^ab^	0.42 ^b^	0.002	0.001
Eggshell thickness (mm)	2 wks	0.39	0.39	0.40	0.39	0.38	0.003	0.653
	4 wks	0.40	0.41	0.41	0.38	0.39	0.004	0.071
	6 wks	0.39	0.38	0.39	0.40	0.39	0.002	0.252
	8 wks	0.40	0.39	0.39	0.39	0.39	0.002	0.141
	0–8 wks	0.39	0.39	0.40	0.39	0.39	0.001	0.198
Relative weight of egg yolk (%)	2 wks	24.79	24.79	24.48	25.23	25.78	0.285	0.707
	4 wks	25.90 ^b^	27.97 ^a^	27.71 ^a^	28.48 ^a^	28.41 ^a^	0.308	0.038
	6 wks	26.34	26.50	26.60	26.13	26.88	0.192	0.815
	8 wks	26.59	27.74	26.93	28.35	26.93	0.225	0.166
	0–8 wks	25.91 ^b^	26.75 ^ab^	26.45 ^ab^	27.05 ^a^	27.00 ^a^	0.130	0.031

Note: Means in a column with different letters differ significantly (*p* < 0.05).

**Table 5:** Effects of different Se sources on egg quality of eggs stored for different times.

Items	Time	Storage Time	Con	0.30 mg/kg SS	0.30 mg/kg Nano-Se	0.30 mg/kg Se-C	0.30 mg/kg Se-Met	SEM	*p*-Value
Albumen height (mm)	2 wks	0 d	10.44	10.74	11.01	11.04	10.79	0.107	0.416
		5 d	5.91	6.11	6.35	6.30	6.26	0.084	0.489
		10 d	4.88 ^b^	5.81 ^a^	6.06 ^a^	5.85 ^a^	5.88 ^a^	0.102	<0.001
		15 d	4.74	5.25	5.55	5.39	5.17	0.107	0.173
	4 wks	0 d	11.01	10.90	10.89	10.66	10.99	0.066	0.498
		5 d	6.38	6.38	6.44	6.47	6.33	0.058	0.961
		10 d	6.11	6.14	6.33	6.34	6.07	0.086	0.811
		15 d	5.37	5.69	5.87	5.68	5.66	0.055	0.061
	6 wks	0 d	11.00	11.51	11.70	11.50	11.39	0.079	0.053
		5 d	6.71	6.92	6.91	7.12	7.23	0.084	0.341
		10 d	6.30	6.59	6.61	6.63	6.42	0.051	0.190
		15 d	5.71	6.01	6.04	6.02	6.00	0.060	0.416
	8 wks	0 d	10.86 ^c^	11.19 ^bc^	11.18 ^bc^	11.63 ^b^	12.19 ^a^	0.115	<0.001
		5 d	7.48	8.04	8.45	8.00	8.24	0.118	0.094
		10 d	6.54	7.04	7.00	6.89	7.07	0.077	0.173
		15 d	6.10	6.31	6.78	6.65	6.61	0.096	0.146
Haugh unit	2 wks	0 d	102.00	103.37	104.50	105.02	103.75	0.450	0.264
		5 d	78.01	78.96	81.60	80.48	79.59	0.642	0.478
		10 d	70.38 ^b^	77.38 ^a^	79.25 ^a^	78.30 ^a^	78.16 ^a^	0.797	< 0.001
		15 d	68.94	73.40	75.67	74.68	73.69	0.863	0.114
	4 wks	0 d	104.24	103.48	103.70	103.07	104.27	0.249	0.526
		5 d	80.96	80.42	81.66	81.21	75.49	1.297	0.583
		10 d	79.03	79.80	80.21	80.82	78.72	0.564	0.793
		15 d	73.47	76.09	77.52	76.65	75.96	0.465	0.059
	6 wks	0 d	103.77 ^b^	105.93 ^a^	106.46 ^a^	106.05 ^a^	105.45 ^a^	0.299	0.026
		5 d	82.67	83.74	83.75	85.31	85.77	0.494	0.267
		10 d	80.05	81.79	81.96	82.07	81.10	0.368	0.407
		15 d	76.05	78.17	78.40	78.38	77.79	0.388	0.280
	8 wks	0 d	102.90 ^c^	104.23 ^bc^	103.62 ^c^	105.84 ^b^	108.03 ^a^	0.462	<0.001
		5 d	86.58	89.66	91.71	89.58	90.49	0.661	0.151
		10 d	80.55	83.87	83.82	83.15	83.90	0.511	0.173
		15 d	77.78	79.46	82.06	81.24	80.43	0.622	0.225

Note: Means in a row with different letters differ significantly (*p* < 0.05).

**Table 6:** Effects of different Se sources on Se content (μg/kg) of yolk.

Items	Time	Con	0.30 mg/kg SS	0.30 mg/kg Nano-Se	0.30 mg/kg Se-C	0.30 mg/kg Se-Met	SEM	*p*-Value
Egg Se Content (μg/kg)	2 wks	161.67 ^b^	425.11 ^a^	396.88 ^a^	430.38 ^a^	431.71 ^a^	24.770	<0.001
	4 wks	153.25 ^b^	335.28 ^a^	320.49 ^a^	337.59 ^a^	348.99 ^a^	18.091	<0.001
	6 wks	106.61 ^c^	306.76 ^b^	342.42 ^a^	353.74 ^a^	340.89 ^a^	21.756	<0.001
	8 wks	107.71 ^c^	385.49 ^b^	391.65 ^ab^	399.14 ^ab^	409.55 ^a^	26.719	<0.001
	0–8 wks	132.31 ^b^	362.86 ^a^	380.21 ^a^	363.16 ^a^	382.78 ^a^	22.263	<0.001

Note: Means in a row with different letters differ significantly (*p* < 0.05).

**Table 7:** Effects of different Se sources on biochemistry and antioxidant enzyme activity of serum and eggs of laying hens.

Items	Indicators	Con	0.30 mg/kg SS	0.30 mg/kg Nano-Se	0.30 mg/kg Se-C	0.30 mg/kg Se-Met	SEM	*p*-Value
Serum	TP (g/L)	38.07	41.61	40.77	41.64	41.94	1.053	0.797
	ALB (g/L)	19.37	19.33	19.25	20.74	20.66	0.426	0.668
	GSH-Px (U/mL)	1574.33 ^b^	2224.48 ^a^	2057.9 ^a^	2258.51 ^a^	2036.42 ^a^	63.449	<0.001
	CAT (U/mL)	1.17	1.34	1.38	1.25	1.50	0.047	0.195
	MDA (nmol/mL)	4.83 ^a^	3.80 ^b^	3.93 ^b^	3.90 ^b^	3.75 ^b^	0.129	0.033
Yolk	TP (g/L)	85.06	93.76	99.40	93.52	97.28	2.485	0.448
	ALB (g/L)	53.43	54.61	53.43	54.61	54.21	0.943	0.991
	GSH-Px (U/mL)	35.43 ^b^	44.93 ^a^	44.68 ^a^	41.90 ^a^	42.25 ^a^	1.041	0.013
	CAT (U/mL)	21.41	22.58	24.75	23.76	23.22	0.532	0.368
	MDA (nmol/mL)	27.50 ^a^	23.25 ^ab^	19.50 ^b^	21.25 ^b^	22.00 ^b^	0.853	0.024

Note: Means in a row with different letters differ significantly (*p* < 0.05).

## Data Availability

The original contributions presented in the study are included in the article, further inquiries can be directed to the corresponding author.
